# Gambling and Gaming: A Comparative Study of Professional Footballers Versus the General Population

**DOI:** 10.3390/sports13020034

**Published:** 2025-01-24

**Authors:** Rafael González-Moret, Isabel Almodóvar-Fernández, María Gimeno, Ana Blanco, Paula Sánchez-Thevenet, Héctor Usó, Gonzalo Haro, Antonio Real-Fernández

**Affiliations:** 1 QSH Research Group, Nursing Predepartmental Unit, Jaume I University, Avenida Vicent Baynat s/n, 12006 Castellón, Spain; moret@uji.es; 2 Research Manager at Villarreal CF SAD, Carrer Miralcamp, 128, 12540 Vila-Real, Spain; 3 School of Health Sciences, Universidad Cardenal Herrera-CEU, C/ Grecia 31, 12006 Castellón, Spain; mgimenom97@hotmail.com (M.G.); a.blanco.alvarado@gmail.com (A.B.); antonio.real@uchceu.es (A.R.-F.); 4 GIRS Research Group, Department of Medicine and Surgery, School of Health Sciences, Universidad Cardenal Herrera-CEU, Calle Grecia 31, 12006 Castellón, Spain; paula.sanchez@uchceu.es; 5 Director of Health Department, Villarreal Football Club, 12540 Villareal, Spain; investigacion@villarrealcf.es; 6 TXP Research Group, Universidad Cardenal Herrera-CEU, Calle Grecia 31, 12006 Castellón, Spain; gonzalo.haro@uchceu.es; 7 Department of Psychiatry, Consorcio Hospitalario Provincial de Castellón, Avenida. del Dr. Clarà, 19, 12002 Castelló de la Plana, Spain

**Keywords:** gambling, gaming, football, male gender

## Abstract

(1) Background: Elite sport can increase vulnerability to developing mental health pathologies. The purpose of the study is to determine the frequency at which these behavioural disorders appear in elite footballers and evaluate their relationship with other addictions. (2) Methods: A cross-sectional study was conducted between November 2020 and January 2022 on 306 participants. The variables of gambling and gaming were studied. The different groups were compared using Chi-squared tests. Probabilities exceeding 95% (*p*-values < 0.05) and residuals results greater than 2 or less than −2 were considered significant. (3) Results: There were significant differences between the two groups in terms of alcohol (*p* < 0.001), tobacco (*p* < 0.001), and cannabis (*p* = 0.016) consumption. We also found differences between those who had a history of a nervous disease (*p* = 0.015). 6.6% of the of football players had a probable diagnosis of a gambling disorder compared to 1% in the general population (*p* = 0.011). Among the pathological and non-pathological cases of gambling in the football population, significant differences were found between those with a salary of EUR 900–1500/month (*p* = 0.027) or a history of a nervous pathology (*p* = 0.021). (4) Conclusions: This study showed that professional football players were vulnerable to mental health pathologies related to gambling.

## 1. Introduction

The World Health Organization (WHO) includes pathological gambling (gambling disorder) among the pathologies that affect people, based on the International Classification of Diseases (ICD 10-ES) ICD10 code F63.0 [1]. Gaming disorder is characterized by an individual having a persistent or recurrent gaming behavior pattern, showing significant impairment in their control over gaming and clinically significant distress for a period of 12 months. Additionally, they prioritize gaming over other interests and daily life activities, using it excessively despite experiencing significant negative consequences that affect various vital areas (social, work/academic, and/or family). Gambling disorder is an impulse control disorder characterized by a compulsive and recurrent need to gamble, despite the negative consequences that may arise as a result.

The global prevalence of gambling disorder in general population is between 0.1% and 6% depending on the region in question. In Spain, the frequency of its appearance has rapidly increased, starting from 2.5% in 2011 and reaching 24% in 2015, due to the legalization of online gambling in Spain in 2012 [2]. Subsequently, video game addiction (gaming disorder), which is known to impact work and academic productivity as well as family relationships [3,4], has also been recognised in the ICD-11 [5], with a prevalence in Spain of 1% in 2022. The prevalence of this disease in elite athletes is 2%, as indicated by some authors [6].

Given the relatively recent recognition of gambling and gaming as pathologies that affect mental health, the objective of this current study was to determine the frequency of appearance of these behavioural disorders among professional league football players, as well as to identify their risk factors and evaluate their relationship with other diseases in the field of addictions in order to provide evidence contributing to their effective prevention and control in elite athletes [6,7,8,9].

In elite athletes, the complexity and high demands of their profession often generate an environment of competitiveness and social isolation that can increase their vulnerability to pathologies related to mental health. In fact, the appearance of disorders such as anxiety and sudden changes in mood is a known problem in this professional group [10]. In turn, this psychopathology is a predisposing factor for mental health from disorders such as substance abuse or behavioural addictions, with a worrying prevalence of gambling [11,12] and gaming [13,14,15] being notable among elite athletes.

It is worth highlighting that in populations with a high socioeconomic level such as elite athletes, the lower economic impact of gaming often delays its diagnosis and therefore, the establishment of early treatment. In addition, professional athletes usually feel they need to respond to demands of greater sporting performance and in so, their risk of substance consumption also synergistically increases [16]. Furthermore, there are other known risk factors in this group such as family disengagement, stress derived from a demanding competition schedule, few external supervision mechanisms, and high levels of economic availability [6]. In an opposite sense and in contrast to everything said, which could serve as an incentive for these athletes, elite athletes often participate directly in the video game industry as actors in products that are of broad commercial interest [17].

## 2. Materials and Methods

### 2.1. Design

A cross-sectional study was conducted between September 2020 and July 2022 with 306 participants divided into two subgroups: footballers from the Spanish Professional League and volunteers from the general population.

### 2.2. Participants

For the professional football player group, non-probabilistic sampling was used for convenience, while a snowball sampling approach was applied in the general population group. An overall recommended sample size of 272 participants (both including professional football players and general population) was calculated using G*Power software (v3.1.9.6) [18], applying an estimated proportion of possible players at risk of developing a gambling/gaming disorder of 8% in the group of professional football players and 1.5% in the general population group, considering a confidence level of 95% (alpha *p*-values less than 0.05), a power of 0.80, and an allocation ratio of 1:2 (1 professional football player: 2 general population).

The inclusion criteria for the professional footballers were: age over 18 years, being a member of a related federation as an active professional in a first division team, having an oral and/or written understanding of the Spanish language, and having signed the informed consent to participate in the study. Participants from the general population met the same inclusion criteria, except the need for them to be a member of a federation or an active football professional.

### 2.3. Gambling and Gaming Variables and Sociodemographic Data

Comprehensive sociodemographic data were collected, including age, sex, marital status, monthly income, and education level. Additionally, participants general health histories, psychiatric conditions, and records of nervous pathologies were documented to ensure a thorough comparison between the groups.

Gambling disorder was determined using the South Oaks Gambling Screen (SOGS) questionnaire, classifying anyone who obtained a score of 4 points or more as a probable pathological gambler. Gaming disorder was determined using the Video Game Addiction Scale for Adolescents (GASA), considering that neurobiological studies have established a limit of 26 years for this age category [19] and given that the average age of the participants in both groups was 27.4 years (SD *=* 9.3) The sociodemographic variables were recorded using a tool from the Addictive Disorders Network (Red de Trastornos Adictivos or RTA in its Spanish acronym) from the multicentre CohRTA cohort [20].

### 2.4. Instruments

The South Oaks Gambling Screen has a Spanish validation and is used by the Directorate General for the Regulation of Gambling (DGOJ) to assess gambling behaviors. This instrument is a common tool to assess GD severity, structured in 20 items that measure cognitive, emotional and other behavior strongly related to gambling problems. The Spanish adaptation of this tool showed good high internal consistency (Cronbach’s alpha *α*  =  0.94) and good test–retest reliability (*r*  =  0.98) [21].

Regarding the GASA scale for assessing video game addiction, it consists of 7 items that evaluate the intensity and frequency of gaming, impulsivity, and peer pressure as influencing factors in problematic gaming. The scoring is direct, meaning that a higher score indicates more problematic gaming behavior. Thus, a score is considered indicative of problematic gaming behavior in relation to video games if there are at least 4 responses of 3 or more, providing a minimum of 15 points [22].

Internal consistency (Cronbach’s alpha) was calculated for both samples. In the general population, the internal consistency values were 0.33 for the SOGS and 0.83 for the GASA. For professional football players the internal consistency values were 0.74 for the SOGS and 0.81 for the GASA.

This study took place from September 2020 to July 2022. Prior to commencing data collection, approval for the project was sought from a first division team. Once their consent was secured, authorization from the ethics committee was obtained. With both approvals in hand, player recruitment commenced. The project was introduced to the rosters of each team, and subsequently, coaches provided a list of players constituting each team. Consequently, each player was assigned a unique code. Football players were then requested to sign the informed consent form.

The questionnaires were transferred to a Microsoft Forms template to facilitate online administration. Two separate distributions were made: one to professional football players and another to the general population who participated in the study voluntarily.

### 2.5. Data Analysis

Data were analysed descriptively using counts and percentages. Differences in the frequency distribution of categorical variables, between study groups, were explored using the Pearson’s Chi Squared test or the Fisher’s exact test, as appropriate. The effect size was calculated using Cramér’s V and were considered small for values > 0.10, moderate for values >0.30 and large for values >0.50 [23]. Univariable logistic regression analyses were carried out to explore the unadjusted effect of independent variables on the likelihood of having a gambling disorder. Multivariable analyses could not be performed due to the small number of participants having a gambling disorder. Values for *p* < 0.05 and results in residuals greater than 2 or less than −2 were considered statistically significant.

Data was managed and analysed using Microsoft Excel^®^, R 4.2.0 software using the Rcmdr 2.7-2 package, and Stata 16.1.

### 2.6. Ethical Factors

In compliance with Law 14/2007, dated 3 July, on biomedical research, all data arising from this study were handled independently of the identity of the study group, in accordance with the principles outlined in:

- The Declaration of Helsinki concerning research involving human subjects (2013).
- Regulation (EU) 2016/679 regarding the protection of natural persons with regard to the processing of personal data and on the free movement of such data.
- Organic Law 3/2018, dated December 5, on the Protection of Personal Data and the guarantee of digital rights.

Moreover, all participants provided written informed consent, and this study was approved by the Ethics Committee for Biomedical Research at the CEU Cardenal Herrera University (CEI19/134) and was registered at ClinicalTrials.org with registration code NCT04842461, as a sub-component of the “Mental Health, addictions and biomarkers in high-performance athletes” study.

## 3. Results

The main sociodemographic characteristics of the participants in the study are shown in Table 1. The average professional football players participants age was 23.5 ± 4.8 years (Range = 40–17), with a higher proportion of male participants also with a higher monthly income and a lower level of education in the professional football player group compared to the participants in the general population.

Table 2 shows the substance consumption habits in the last 30 days and pathological and mental health history of the population studied (football players and general population). A total of 6.6% of the of professional football players had a probable diagnosis of a gambling disorder compared to 1% in the general population. By sex, a gambling disorder appeared more frequently in males among the football player group, while the opposite was true in the general population group. Regarding gaming, no significant differences were observed between the professional football players compared to the general population, with 6.5% of the former and 4% of the latter presenting a gaming disorder. All the cases of gaming disorder among the football players were male (10.8% of the men in this group) and in the case of the general population, 9.6% and 2% of the men and women, respectively, presented a gaming disorder.

In terms of gambling, given that differences were found between the professional football players and the general population, we deepened the analysis by grouping all the cases of gambling disorder and comparing them to all the participants with no gambling disorder to determine possible factors related to this behavioural addiction. The results of this analysis are shown in Table 3 which indicates that there were no sociodemographic differences between these groups except in the participants who received an income between EUR 900 and EUR 1500 per month, in which a gambling disorder was more frequent. Finally, a history of a dual diagnosis or polysubstance use was more common among participants with a gambling disorder.

Univariable logistic regression analyses indicated that football players with an average monthly income of EUR 900–1500 were about nine times more likely to have a gambling disorder than those earning less than EUR 450 per month (*p* = 0.049) (Table 4).

## 4. Discussion

The prevalence of gambling disorder is notably higher among professional football players compared to the general population with a small effect size (V = 0.15). This finding is significant as it sheds light on a behavioral health issue that may be exacerbated within the context of professional sports environments. The higher prevalence of gambling disorder among male football players underscores the need for targeted interventions and support systems tailored to the specific needs of athletes, particularly those at risk of developing addictive behaviors. No significant differences in gaming disorder prevalence were observed between football players and general population. It is noteworthy that all cases of gaming disorder among football players were male. This gender disparity in gaming disorder prevalence warrants further investigation into the underlying factors contributing to excessive gaming behaviors among male athletes.

The results highlight the need to monitor the profiles that have shown a higher propensity to develop this condition and to conduct future research on preventive methods for this addiction.

These findings regarding sex confirm the proposals by Vinberg [24] and Håkansson [25], and also support the data from the study carried out by the Spanish General Directorate of Gambling Regulation [26], which showed that men were more greatly impacted by this behavioural disorder. In turn, these results also demonstrate that professional football players are vulnerable to mental health pathologies such as gambling addictions, adding to previously reported evidence from different studies that there is a greater risk of gambling disorder among elite athletes [27,28]. In addition to being male, the level of income and a history of pathologies related to mental health, with the latter two factors having a moderate effect size (V = 0.36 and V = 0.35 respectively), were also factors that our findings suggested were associated with the possibility of having a gambling disorder in our study population (elite players), thereby coinciding with the report by Dominguez et al. [19]. To deepen our knowledge of this subject, studies analysing the problem of gambling disorders in different types of elite athletes would need to be carried out to elucidate whether the risk is greater in individual or group sports or if it is affected by other variables.

In this current work, the prevalence of the consumption of toxic substances or appearance of risky behaviours seemed to be higher in the professional athlete group, as also previously shown by Huang and colleagues [29]. Participants with a history of dual diagnosis or polysubstance use were more likely to exhibit a gambling disorder. This finding underscores the complex interplay between behavioral addictions and co-occurring mental health conditions, emphasizing the importance of integrated treatment approaches that address both substances use disorders and behavioral addictions concurrently. Prior studies have shown that the consumption of substances such as tobacco, cannabis, or alcohol are significantly related to gambling disorder [30,31] and have suggested that the impulsive–compulsive personality trait is associated with a greater risk of presenting this pathology. We were unable to find any previous work that had related these risk factors in professional football players. Football players reported lower alcohol and tobacco consumption, and a lower prevalence of nervous diseases compared to the general population. These findings suggest that while football players are vulnerable to gambling disorder, certain protective factors related to their professional lifestyle may mitigate other mental health risks, the effect size being moderate for tobacco (V = 0.33) and almost moderate for alcohol (V = 0.29).

Our findings showed that the prevalence of gaming disorder in both professional athletes and the general population was similar, although it was slightly higher among the professional football players. According to Corey et al. [32], the number of hours spent on gaming and the social and mental health repercussions this can entail can be harmful to the health of the individual, who may be seeking to satisfy needs for autonomy, competence, and relationships not satisfied in their everyday lives. Of note, football professionals are subjected to high demands that can cause them to be more competitive which could lead them towards more isolated social situations, perhaps resulting in behavioural problems related to mental health. Some of the variables that may influence this problem have been analysed in this study, as mental health is one of the most pressing challenges currently facing elite sport [8,33,34].

Changes in mood and anxiety are especially important in relation to mental health from substance use disorders, gambling abuse or video game addiction. Importantly, the repercussions of these behaviours are not as evident as they are for substance use disorders and so their detection can be delayed by longer. Nonetheless, the psychological and emotional consequences are serious and can affect not only the family relationships and psychological aspects of the individual, but also the course of their professional career as an athlete. Indeed, behavioural dysfunctions, depression, attention deficit, low self-esteem, or social anxiety are all related to gaming disorder [35], a problem that was detected at a slightly higher frequency among general population in our study when compared to the elite football players.

Finally, money holds a pivotal position in gambling, and gaining insight into the varied perspectives of gamblers regarding it could prove advantageous for both preventing and addressing gambling-related issues [36]. The analysis in our study indicates a relationship between moderate income levels (EUR 900–EUR 1500 per month) and a higher prevalence of gambling disorder, as the effect size has been shown to be strong (odds ratio = 8.8). This connection suggests that financial status may influence susceptibility to gambling addiction, highlighting the need for targeted interventions addressing financial literacy and responsible gambling practices among individuals with moderate incomes.

### Strengths and Limitations

The aim of this study is to highlight how gambling affects elite athletes, specifically footballers in the first division of the Spanish League. Being aware of this, clubs can make decisions to improve the situation, reducing or trying to eliminate this problem.

Through these experiences, we have become aware of how gender, race, and socioeconomic status influence training opportunities and outcomes.

This study analyzed, for the first time, the gaming and gambling of a professional soccer team belonging to the first and second divisions. In addition, the inclusion of both a women’s and a men’s team made it possible to compare the results by sex.

However, it is important to note that this study had some limitations such as the fact that the sampling was non-probabilistic, which could imply that there was a possible selection bias. Although data were collected from different categories and genders, using only one club may present a limitation when extrapolating the results to other Spanish league clubs or clubs from other countries. Furthermore, there were notable socioeconomic differences between the two study groups, especially in terms of their education levels and salaries. Nevertheless, the need to establish mechanisms to prevent behavioural disorders such as gambling and gaming disorder among elite football players was evident in this work. Protocols for the early detection of these complex mental health pathologies in these athletes must also be developed that consider their history of nervous pathologies as well as various risk factors such as a high income level that can increase their vulnerability to these disorders. The study adopts an approach consistent with the medical model of mental health, which offers strengths in identifying risk factors and vulnerabilities. However, this approach may be limited as it does not incorporate other biopsychosocial models.

The Cronbach’s alpha value of 0.33 for the SOGS questionnaire administered to the general population indicates low consistency, which could impact the scores and effect sizes in comparisons involving the general population. This may be due to the fact that, since the general population is more diverse in terms of sociodemographic characteristics, behaviors, and attitudes towards gambling, greater variability could have been introduced in the questionnaire responses, thereby reducing internal consistency. This could be improved by increasing the sample size.

## 5. Conclusions

The results of this study underscore the multifaceted nature of behavioural health challenges within both athletic and general populations. It will be very important to prevent future problems with gaming and gambling disorders, that professional football players, especially men, are more vulnerable to gambling disorders, and a comprehensive approach that considers both financial and mental health factors is needed to prevent and treat these issues. This could be linked to the competitive environment and the pressures of elite sports, highlighting the need for targeted interventions for this group. Additionally, while gaming disorder is similar among football players and the general population, its prevalence among men suggests a particular phenomenon that warrants further investigation.

The prevalence of gambling disorders in our sample was rather low. While this not a limitation of our study aims or conclusions, it did limit the possibility of looking at the factors that may be associated with having this disorder. Future studies looking at this will need to have a much larger sample size. Given that the number of football players that a single football team can have will always be limited, several football teams will need to be included and, therefore, analyses will need to account for the clustered structure of the data.

## Figures and Tables

**Table 1:** Sociodemographic characteristics of the population studied (N = 306).

	Professional Football Players (*n* = 108)		General Population (*n* = 198)		Cramer’s V	*p*-Value * Adjusted Residues
	*n*	(%)	*n*	(%)		
**Sex**					0.33	**<0.001**
Men	65	(60.2)	52	(26.3)		5.8/−5.8
Women	43	(39.8)	146	(73.7)		−5.8/5.8
**Marital status**					0.09	ns
Single	91	(84.3)	151	(76.3)		
Married	15	(13.9)	40	(20.2)		
Divorced/Separated	2	(1.8)	6	(3.5)		
**Living arrangements**					0.07	ns
Alone	11	(10.2)	12	(6.1)		
Shared accommodation	97	(89.8)	186	(93.9)		
**Education level**					0.35	**<0.001**
Uneducated	**14**	**(12.9)**	**0**	**(0.0)**		**5.2/−5.2**
Primary education	72	(66.7)	111	(56.1)		1.8/−1.8
Graduate level education	**22**	**(20.4)**	**87**	**(43.9)**		**−4.1/4.1**
**Monthly income**					0.49	**<0.001**
<EUR 450	**23**	**(21.3)**	**94**	**(47.5)**		**−4.5/4.5**
EUR 450–900	**22**	**(20.3)**	**14**	**(7.1)**		**3.5/−3.5**
EUR 900–1500	23	(21.3)	48	(24.2)		−0.6/0.6
EUR 1500–2100	**8**	**(7.4)**	**30**	**(15.2)**		**−2.0/2.0**
EUR 2100−2700	2	(1.9)	8	(4.0)		−1.0/1.0
EUR 2700−3600	2	(1.9)	2	(1.0)		0.6/−0.06
>EUR 3600	**28**	**(25.9)**	**2**	**(1.0)**		**7.0/−7.0**

* The frequency distributions of variables are compared using the Pearson’s Chi Squared test or the Fisher exact test, as appropriate. ns = no significance.

**Table 2:** The substance consumption habits and pathological and mental health history of the population studied (N = 306).

		Football Players (*n* = 108)		General Population (*n* = 198)		Cramer’s V	*p*-Value * Adjusted Residues
		*n*	(%)	*n*	(%)		
**Consumption in the last 30 days**	**Alcohol**					0.29	**<0.001**
	Yes	40	(37.0)	133	(67.2)		−5.1/5.1
	No	68	(63.0)	65	(32.8)		5.1/−5.1
	**Tobacco/** **Nicotine**					0.33	**<0.001**
	Yes	0	(0.0)	50	(25.3)		−5.7/5.7
	No	108	(100.0)	148	(74.7)		5.7/−5.7
	**Cannabis**					0.14	**0.016**
	Yes	0	(0.0)	10	(5.1)		−2.4/2.4
	No	108	(100.0)	188	(94.9)		2.4/−2.4
	**Opiates**					0.06	ns
	Yes	0	(0.0)	2	(1.0)		
	No	108	(100.0)	196	(99.0)		
	**Sedatives**					0.10	ns
	Yes	1	(0.9)	9	(4.5)		
	No	107	(99.1)	189	(95.5)		
	**Other drugs**					0.13	**0.037**
	Yes	7	(6.5)	3	(1.5)		2.3/−2.3
	No	101	(93.5)	195	(98.5)		−2.3/2.3
**History of general pathologies**						0.01	ns
Yes		27	(25.0)	47	(23.7)		
No		81	(75.0)	151	(76.2)		
**Psychiatric history**						0.06	ns
Yes		0	(0.0)	2	(1.0)		
No		108	(100.0)	196	(99.0)		
**History of a nervous disease**						0.14	**0.015**
Yes		4	(3.7)	24	(12.1)		−2.4/2.4
No		104	(96.3)	174	(87.9)		2.4/−2.4
**Mental health specialist visit**						0.11	ns
Yes		36	(33.3)	88	(44.4)		
No		72	(66.7)	110	(55.6)		
**SOGS**						0.15	**0.011**
Gambling disorder		7	(6.6)	2	(1.0)		2.7/−2.7
No gambling disorder		99	(93.4)	190	(99.0)		−2.7/2.7
**GASA**						0.05	ns
Gaming disorder		7	(6.5)	8	(4.0)		
No gaming disorder		101	(93.5)	190	(96.0)		

* The frequency distributions of variables are compared using the Pearson’s Chi Squared test or Fisher’s exact test, as appropriate. ns = No significance.

**Table 3:** Distribution of the sociodemographic characteristics, history of psychopathologies, and mental health care of the study participants according to the presence or absence of a gambling disorder.

	No Gambling Disorder (*n* = 99)		Gambling Disorder (*n* = 7)		Cramer’s V	*p*-Value * Adjusted Residues
	*n*	(%)	*n*	(%)		
**Sex**					0.14	ns
Men	57	(57.6)	6	(85.7)		
Women	42	(42.4)	1	(14.3)		
**Marital status**					0.12	ns
Single	82	(82.8)	7	(100.0)		
Married	15	(15.2)	0	(0.0)		
Divorced/Separated	2	(2.0)	0	(0.0)		
**Living arrangements**					0.03	ns
Alone	10	(10.1)	1	(14.3)		
Shared accommodation	89	(89.9)	6	(85.7)		
**Education level**					0.14	ns
Uneducated	13	(13.2)	1	(14.3)		
Primary education	64	(64.6)	6	(85.7)		
Graduate level education	22	(22.2)	0	(0.0)		
**Monthly income**					0.36	**0.027**
<EUR 450	23	(23.2)	0	(0.0)		1.4/−1.4
EUR 450–900	21	(21.2)	1	(14.3)		0.4/−0.4
EUR 900–1500	**17**	**(17.2)**	**5**	**(71.4)**		**−3.4/3.4**
EUR 1500–2100	7	(7.1)	1	(14.3)		−0.7/0.7
EUR 2100−2700	2	(2.0)	0	(0.0)		0.4/−0.4
EUR 2700−3600	1	(1.0)	0	(0.0)		0.3/−0.3
>EUR 3600	28	(28.3)	0	(0.0)		1.6/−1.6
**Alcohol consumption in last 30 days**					0.12	ns
Yes	37	(34.3)	1	(57.1)		
No	62	(65.7)	6	(42.9)		
**Sedatives consumption in last 30 days**					0.03	ns
Yes	1	(1.0)	0	(0.0)		
No	98	(99.0)	7	(100.0)		
**Other drugs consumption in last 30 days**					0.08	ns
Yes	6	(6.1)	1	(57.1)		
No	93	(93.9)	6	(42.9)		
**History of general pathologies**					0.03	ns
Yes	23	(23.2)	2	(28.6)		
No	76	(76.8)	5	(71.4)		
**History of a nervous disease**					0.35	**0.021**
Yes	2	(2.0)	2	(28.6)		−3.6/3.6
No	97	(98.0)	5	(71.4)		3.6/−3.6
**Mental health specialist visit**					0.14	ns
Yes	31	(31.3)	4	(57.1)		
No	68	(68.7)	3	(42.9)		

* The frequency distributions of variables are compared using the Pearson’s Chi Squared test or the Fisher’s exact test, as appropriate. ns= No significance.

**Table 4:** Likelihood for developing a gambling disorder amongst football players. Univariable logistic regression.

	Odds Ratio (95% Confidence Interval)	*p*-Value
**Sex**		
Men	3.4 (0.8, 13.9)	0.087
Women (reference)		
**Marital status**		
Single (reference)		
Married	0.6 (0.1, 5.2)	0.670
Divorced/Separated	4.2 (0.5, 38.1)	0.205
**Living arrangements**		
Alone		
Shared accommodation	0.3 (0.1, 1.4)	0.114
**Education level**		
Uneducated (reference)	NV	NV
Primary education	NV	NV
Graduate level education	NV	NV
**Monthly income**		
<EUR 450 (reference)		
EUR 450–900	6.8 (0.6, 77.6)	0.122
EUR 900–1500	8.8 (1.01, 76.8)	**0.049**
EUR 1500–2100	0	
EUR 2100−2700	0	
EUR 2700−3600	0	
>EUR 3600	4.3 (0.3, 70.9)	0.308
**Alcohol consumption in last 30 days**		
Yes (reference)		
No	2.7 (0.7, 11.1)	0.163
**Sedatives consumption in last 30 days**		
Yes (reference)	NV	NV
No	NV	NV
**Other drugs consumption in last 30 days**		
Yes (reference)		
No	0.2 (0, 2)	0.180
**History of general pathologies**		
Yes (reference)		
No	1.3 (0.2, 10.6)	0.812
**History of a nervous disease**		
Yes (reference)		
No	0.8 (0.1, 7)	0.871
**Mental health specialist visit**		
Yes (reference)		
No	0.5 (0.1, 2.1)	0.367

Ref: reference category; NV: not enough data/variability to perform the analyses.

## Data Availability

The data that support the findings of this study are available from the corresponding author, I.A.-F., upon reasonable request.
